# Reduced spinopelvic mobility does not correlate with knee flexion deformity in patients undergoing total knee arthroplasty

**DOI:** 10.1002/ksa.70047

**Published:** 2025-09-09

**Authors:** Lorenz Pichler, Rauf Alizada, Lea M. S. Cordes, Kerem Basarir, Asim Kayaalp, Reha Tandogan

**Affiliations:** ^1^ Department of Orthopedics and Trauma‐Surgery Medical University of Vienna Vienna Austria; ^2^ Charité ‐ Universitätsmedizin Berlin Center for Musculoskeletal Surgery Berlin Germany; ^3^ Çankaya Hospital for Orthopedic Care Ankara Turkey; ^4^ Charité ‐ Universitätsmedizin Berlin Berlin Institute of Health (BIH) ‐ Julius Wolff Institute (JWI) Berlin Germany

**Keywords:** global sagittal alignment, knee flexion deformity, knee phenotypes, robotics, spinopelvic mobility, total knee arthroplasty

## Abstract

**Purpose:**

The aim of this study was to evaluate the impact of reduced spinopelvic mobility (SM) on knee flexion deformity (KFD) in patients undergoing total knee arthroplasty (TKA).

**Methods:**

A retrospective analysis on 213 patients (271 knees) undergoing robotic‐assisted primary TKA was conducted. Sagittal spinopelvic alignment (SSA) parameters—sacral slope (SS), pelvic incidence (PI), and pelvic tilt (PT)—were measured on lateral standing and sitting spinopelvic radiographs. Patients were stratified according to established SM classifications: standing‐sitting difference in SS ≥ 10° versus < 10°, PT ≥ 20° versus < 20°, and Dorr's classification (DC). KFD was assessed intraoperatively using a robotic‐assisted surgical platform after placement of arrays: first in the native state (KFDb), and again after osteophyte removal and maximum manual correction (KFDa). Correlations between standing‐sitting changes in SSAs and KFDb/KFDa, as well as differences in mean KFDb/KFDa between SM groups, were analysed.

**Results:**

The mean differences between standing and sitting was 11.3° (SD 9.1) for SS and 6.9° (SD 9.8) for PT. Based on SM classifications, 129 knees (48%) showed a SS difference < 10°, 30 knees (11%) a PT difference ≥ 20°, and DC categorised 131 (48%) as normal, 64 (24%) as stuck standing, 74 (27%) as stuck sitting, and 1% as kyphotic. The mean KFDb and KFDa were 5° (SD 6) and 2° (SD 2), respectively. No significant correlations were found between standing‐sitting changes in SSAs and KFDb or KFDa (correlation coefficients < 0.1 for all). No significant differences in mean KFDb or KFDa were observed across SM classification groups (*p* > 0.2 for all).

**Conclusion:**

Spinopelvic mobility does not correlate with intraoperative knee flexion deformity in patients undergoing TKA suggesting that the increased knee flexion found among these patients is a dynamic rather than a permanent compensatory mechanism.

**Level of Evidence:**

Level III.

**Abbreviations**
ANOVA: analysis of variance
AV: acetabular version
CT: computer tomography
DC: Dorr's classficiation
KFD: knee flexion deformity
KFDa: knee flexion deformity after bone cuts and removal of osteophytes
KFDb: knee flexion deformity before bone cuts and removal of osteophytes
KOA: knee osteoarthitis
PI: pelvic incidence
PROMS: Patient‐Reported Outcome Measures
PT: pelvic tilt
RASP: robotic‐assisted surgical platform
ROM: range of motion
rTKA: robotic‐assisted TKA
SD: standard deviation
SM: spinopelvic Mobility
SS: sacral slope
SSA: sagittal spinopelvic alignment
TKA: total knee arthroplasty

## INTRODUCTION

Sagittal spinopelvic alignment (SSA) and mobility (SM) have become critical factors in total hip arthroplasty [31]. However, recent investigations suggest that their influence extends beyond the hip joint, also affecting the biomechanics of the knee joint. Specifically, lumbar spinal degeneration—characterised by reduced SM and altered SSA—has been associated with increased knee flexion on static sagittal radiographs [19, 20, 32]. Yet, it remains unclear whether lumbar spinal degeneration acts as a causative factor for increased knee flexion and potential knee flexion deformity (KFD), or whether it is, in part, a consequence of such deformity [5].

Several studies have investigated the effect of total knee arthroplasty (TKA) and the associated correction of knee flexion deformity on sagittal spinal alignment, but findings remain contradictory. While some report no significant changes in SSA parameters after TKA, others have observed reduced anterior pelvic tilt (PT) and sacral slope (SS), in patients postoperatively, compared to those with untreated knee osteoarthritis and flexion deformity [21, 24, 26].

Importantly, many of these studies rely on single radiographs, which fail to capture the dynamic nature of spinopelvic mobility. Since spinopelvic mobility is inherently a functional concept, its assessment requires evaluation of changes in sagittal spinopelvic alignment across different positions, such as standing and sitting [23]. Furthermore, single radiographs only represent momentary representations of alignment and may only reflect temporary compensatory mechanisms—such as increased knee flexion to offset a pathological SSA and prevent anterior imbalance—rather than fixed deformities like true knee flexion deformity [25].

Therefore, the present study aims to investigate the correlation between spinopelvic mobility—defined as the change in SSA parameters between standing and sitting radiographs—and knee flexion deformity, assessed dynamically using a robotic‐assisted surgical platform (RASP) during TKA. The hypothesis put forward is that reduced spinal mobility, as classified by established criteria, correlates with increased KFD.

## METHODS

### Patients

All patients undergoing an image based robotic‐assisted TKA (rTKA) at a single institution between August 2022 and July 2024 were screened for inclusion. Inclusion criteria were defined as follows: Primary uni‐ or bilateral rTKA using the MAKO RASP (Stryker Corporation, Kalamazoo, MI, US), for the treatment of knee osteoarthritis; availability of standing and sitting lateral spinopelvic radiographs; availability of intraoperative data on knee range of motion (ROM) in the native state and after removal of osteophytes with maximum manual correction, and written patient consent. Exclusion criteria included post‐traumatic or post‐infectious arthritis, previous surgery to the affected and/or contralateral knee, hip and/or spine, and patients undergoing revision or unicompartmental knee arthroplasty.

A total of 221 patients met the inclusion criteria. Of these, eight patients (10 knees) were excluded due to insufficient image quality, resulting in a final cohort of 213 patients with 271 TKA's (155 patients with unilateral TKAs and 58 with bilateral TKAs). Patient demographics and knee phenotypes according to the Coronal Plane Alignment of the Knee (CPAK)‐and Functional Knee Phenotypes (FKP) Classifications are presented in Table 1 and Supporting Information 1 [11, 18].

**Table 1:** Patient demographics.

	Overall	Unilateral TKA	Bilateral TKA
Patients, *N*	213	155	58
Age^a^	70.5 (33–90)	71.7 (33–90)	68.8 (53–83)
Female/male, %	83/17	78/22	95/5

Abbreviation: TKA, total knee arthroplasty.

^a^ Median (range).

### Radiographic parameters

**Figure 1:**
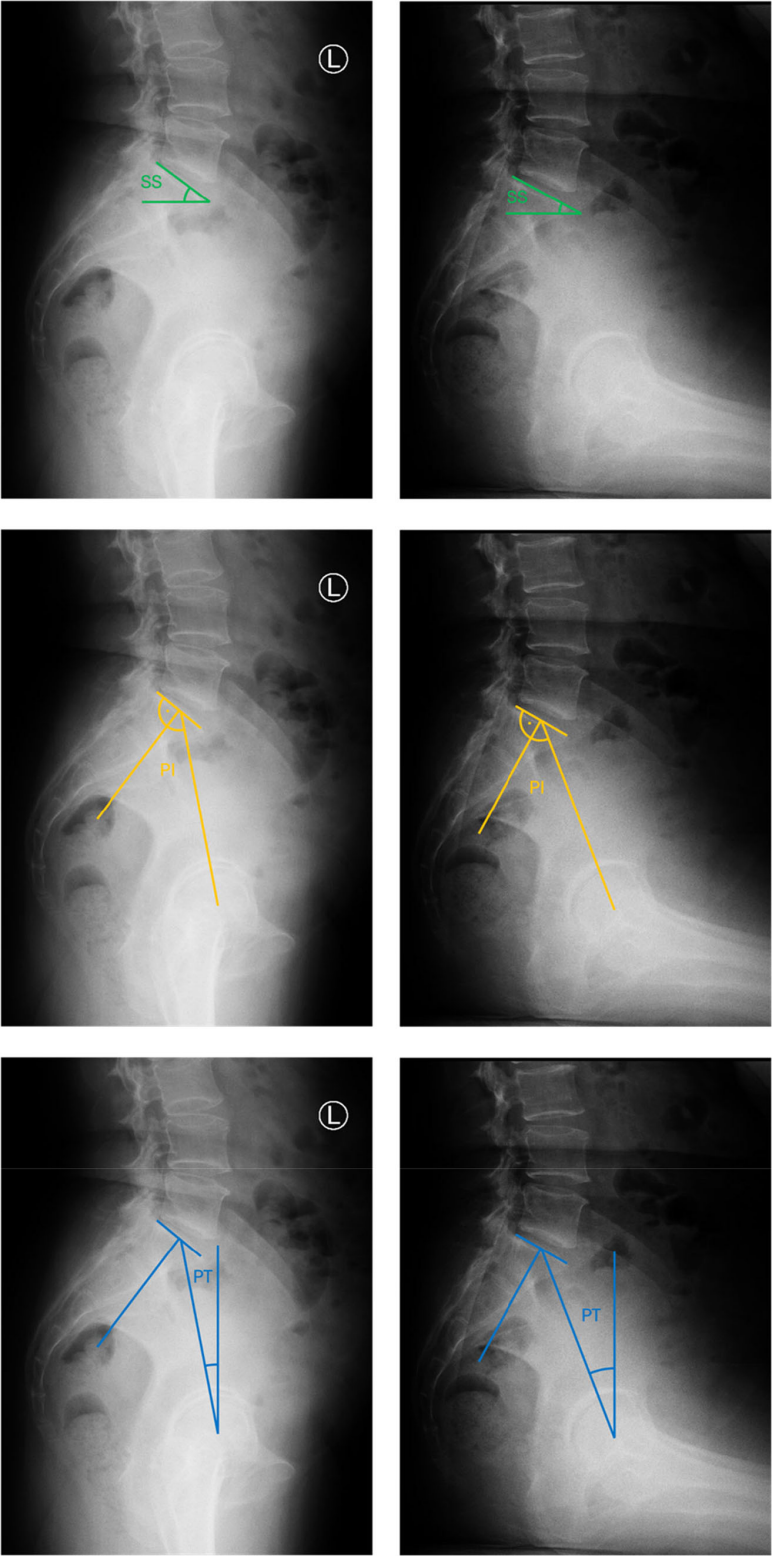
Measurements of sagittal spinopelvic alignment (SSA) parameters standing and sitting. Top row—Sacral slope (SS). Middle row—Pelvic incidence (PI). Bottom row—Pelvic tilt (PT).

The following established parameters of SSA were measured on lateral standing and sitting spinopelvic radiographs: SS, pelvic incidence (PI) and PT. SS was defined as the angle between the superior endplate of S1 and the horizontal. PI was reported as the angle between the line perpendicular to the sacral endplate at its midpoint and the line connecting this point to the femoral head axis. PT was measured as the angle between the vertical and the line connecting the midpoint of the sacral endplate to the femoral head axis. Measurements of SSAs are illustrated in Figure 1. In addition, acetabular version (AV) was measured on preoperative CT scans recorded in supine position. All parameters were measured in degrees with an accuracy of one decimal point based on the PACS system used.

Patients were stratified according to the following classifications of SM: standing‐sitting SS change ≥ 10° versus < 10° [8], standing‐sitting PT change ≥ 20° versus < 20° [16], and Dorr's classification (DC [normal, stuck standing, stuck sitting, and kyphotic]) [1]. Reduced spinopelvic mobility was defined as a standing‐sitting change in SS < 10° and/or a standing‐sitting change in PT < 20°. All radiographic measurements were performed independently by two observers. Interobserver reliability ranged from moderate (0.4–0.6) to good (0.61–0.8) [17]. The mean of both measurements was used for further analysis.

### Surgery and intraoperative data

**Figure 2:**
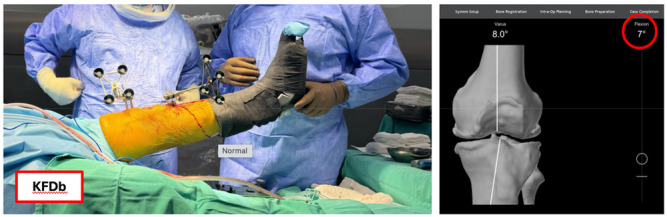
Intraoperative measurement of knee flexion deformity before bone cuts and removal of osteophytes (KFDb) (left) using the robotic‐assisted surgical platform (RASP) (right).

All procedures were performed with the patient in the supine position under regional anaesthesia (spinal or combined spinal‐epidural), without the use of a tourniquet, and via a standard medial parapatellar approach. All patients received a Stryker Triathlon® cemented total knee implant through a standard medial parapatellar approach. Arthrotomy was followed by placement of checkpoints, fixation of femoral and tibial arrays with Schanz screws, determination of hip center, bone registration and verification. Passive knee flexion deformity (KFD) was measured twice by the robotic system with an accuracy of 1°. The first measurement (KFDb) was performed in the native state by passively lifting the operative leg at the heel, allowing the robotic system to record the knee extension angle without any corrective force (Figure 2). The second measurement was performed after section of the anterior cruciate ligament (if present), removal of tibial and femoral osteophytes and limited medial release up to the deep medial collateral ligament followed by recording of knee extension in maximal manual correction (KFDa).

All surgeries were performed by a team of three senior arthroplasty surgeons, each with a minimum of 15 years of experience in total knee arthroplasty.

### Statistics

Demographic data were analysed, and means, standard deviations, medians, and ranges were reported as appropriate. Correlations between changes in SSA parameters between standing and sitting and both KFDb and KFDa were evaluated using Pearson's correlation coefficient.

Subgroup analyses regarding differences in mean KFDb and KFDa between groups stratified by SM classifications (SS and PT) were assessed using Wilcoxon tests, while comparisons according to Dorr's classification and CPAK‐type were conducted using Kruskal‐Wallis. To further examine the relationship between CPAK types and KFDb/KFDa, a Dunn post hoc test was used. However, due to the limited sample size for CPAK types VII and IX, the Dunn test was only applied to CPAK types I and III.

Post hoc power analyses revealed a statistical power of 0.99 for bivariate correlations (two‐tailed, Cohen's *d* = 0.30, *α* = 0.05); 0.06 for Wilcoxon tests comparing SS and PT groups (calculated *r* = 0.03–0.06, *α* = 0.05); 0.10–0.24 for Kruskal–Wallis tests comparing Dorr classifications (calculated Cohen's *f* = 0.054–0.10, *α* = 0.05); and 0.34–0.83 for Kruskal–Wallis tests comparing CPAK types (calculated Cohen's *f* = 0.118–0.208, *α* = 0.05), based on the current sample size. All analyses were performed using Microsoft Excel for Mac (Version 16.96) and R (Version 4.3.2).

### Ethical aspects

Written informed consent was obtained from all patients for use of their anonymized demographic, radiological & intra‐operative robotic data. Institutional Review Board Approval was obtained from the local Ethics Committee for Clinical Studies of (No: 2/2024, 11 November 2024).

## RESULTS

A change in SS of ≥10° was observed in 142 cases (52.4%), while 129 cases (47.6%) had a change <10°. For PT, 30 cases (11%) showed a difference ≥20°, and 241 cases (89%) <20%. According to Dorr's classification, 131 knees (48.3%) were categorised as normal, 64 (23.6%) as stuck standing, 74 (27.3%) as stuck sitting, and 2 (0.7%) as kyphotic. Individual parameters of SSA for standing and sitting are presented in Table 2.

**Table 2:** SSA parameters and correlation of change with KFDb and KFDa.

	Standing	Sitting	Change	KFDb	KFDa
Sacral slope (SS), °	35.1 (8.5)	23.8 (9.8)	11.3 (9.1)	0.048^a^	−0.006^a^
Pelvic incidence, °	53.0 (10.3)	54.1 (11.2)	−1.0 (6.2)	0.024^a^	0.063^a^
Pelvic tilt, °	20.8 (8.8)	27.7 (10.2)	−6.9 (9.6)	−0.043^a^	−0.015^a^
Acetabular version, °^b^	17.9 (5.2)	‐	‐	−0.097^a^	−0.068^a^

*Note*: Values represent means with standard deviations in brackets if not stated otherwise.

Abbreviations: CT, computer tomography; KFDa, knee flexion deformity after bone cuts and removal of osteophytes; KFDb, knee flexion deformity before bone cuts and removal of osteophytes.

^a^ Pearson correlation coefficient.

^b^ Acetabular version was measured CT scan in supine position.

The overall mean KFDb was 5° (SD 6). For unilateral TKA, KFDb was 4.7° (SD 6.1), and for bilateral TKA, 4.5° (SD 5.6), with a mean left/right difference of 3° (SD 2.3). KFDa showed an overall mean of 2° (SD 2), with values of 1.9° (SD 2.5) for unilateral and 1.8° (SD 2.5) for bilateral TKA, and a mean left/right difference of 2.8° (SD 1.8).

Correlation coefficients between changes in SSA parameters as well as AV and both KFDb and KFDa were reported between 0.01 and 0.06 for all comparisons, indicating a small effect size according to Cohen's classification [3] and no correlations (see Table 2). Additionally, subgroup analyses revealed no statistically significant differences in KFDb or KFDa between patients with SS differences ≥ 10° versus < 10°, PT differences ≥ 20° versus < 20°, and across Dorr's classification groups. However, a statistically significant difference in mean KFDb was observed across preoperative CPAK types (*p* = 0.01; see Table 3). Post hoc Dunn testing showed that CPAK type III had significantly higher KFDb compared to CPAK type I (*p* = 0.015), while no significant difference was found for KFDa (*p* = 0.223).

**Table 3:** Differences between KFDb and KFDa according to subgroups.

	KFDb	*p*‐value	KFDa	*p*‐value
Difference SS standing/sitting, °				
≥10 (*n* = 129)	4.6 (6.0)		1.9 (2.3)	
<10 (*n* = 142)	4.5 (5.8)	0.523^a^	1.8 (2.6)	0.678^a^
Difference PT standing/sitting, °				
≥20 (*n* = 30)	5.7 (5.5)		1.9 (1.8)	
<20 (*n* = 241)	4.5 (5.9)	0.305^a^	1.8 (2.6)	0.402^a^
Dorr's classification				
Normal (*n* = 131)	4.5 (5.9)		1.8 (2.4)	
Stuck standing (*n* = 64)	4.7 (5.6)		2.1 (2.9)	
Stuck sitting (74)	4.6 (6.0)		1.7 (2.4)	
Kyphotic (*n* = 2)	7.5 (5.0)	0.855^b^	0 (0)	0.447^b^
CPAK‐Type				
I (*n* = 227)	4.7 (5.9)		1.7 (2.4)	
III (*n* = 36)	2.7 (5.0)		2.1 (2.4)	
VII (*n* = 6)	7 (3.2)		1.9 (2.6)	
IX (*n* = 2)	16 (9.9)	0.01^b^	7 (7)	0.291^b^

*Note*: Values represent means with standard deviations in brackets if not stated otherwise.

Abbreviations: CPAK, coronal plane alignment of the knee; KFDa, knee flexion deformity after bone cuts and removal of osteophytes; KFDb, knee flexion deformity before bone cuts and removal of osteophytes.

^a^ Wilcoxon test.

^b^ Kruskal–Wallis test.

## DISCUSSION

The most important finding of this study was that spinopelvic mobility parameters do not correlate with knee flexion deformity when measured intraoperatively by a robotic assisted platform in patients undergoing primary TKA. Therefore, the hypothesis of this study that SSA parameters correlate with intraoperative knee flexion deformity had to be rejected.

This challenges the assumption that reduced spinopelvic mobility directly contributes to knee flexion deformity. In previous literature, two hypotheses have been proposed for the increased static knee joint flexion observed in patients with pathological sagittal spinal alignment and reduced spinopelvic mobility [20]:

A. a dynamic and reversible compensatory mechanism in response to global sagittal malalignment—serving to prevent forward stooping, or
B. secondary to knee osteoarthritis, which may trigger pathological SSA and reduced spinopelvic mobility rather than result from them.

Hypothesis A is supported by findings from Kim et al., who investigated global sagittal alignment before and after TKA using EOS full body radiographs [14]. They reported a significant negative correlation between one‐year postoperative knee flexion deformity and lumbar lordosis, suggesting that increased standing and walking knee flexion prevails even in patients where the flexion deformity was corrected through TKA. This led them to hypothesises that: *“… the axial and lower limb alignments interact to aid in balance …”*. Given that the present study found no correlation between reduced SM and intraoperative non‐weightbearing KFD, it is implied that the increased KFD observed in patients with reduced lumbar lordosis by Kim et al. may be muscularly controlled or potentially a consequence of weight‐bearing measurement conditions.

Hypothesis B is supported by the findings of Oshima et al., who approached the knee‐hip‐spine relationship from the bottom up, investigating the influence of the knee on spinal alignment [21]. They reported improvements in the sagittal vertical axis—a parameter of static posture—following TKA and the associated reduction in knee flexion deformity.

Regardless of directionality, the interconnected nature of the axial and peripheral skeleton has long been recognised. As early as 1994, Jean Felix Dubousset described the “cone of economy” concept, positing that degenerative spinal changes requiring postural adaptation are balanced by compensatory changes in the peripheral skeleton [4]. However, the results of the present study and those of Kim et al. [14] and Oshima et al. [21] suggest this compensatory mechanism may be a two‐way rather than a one‐way street, with peripheral changes (e.g., from osteoarthritis) influencing spinal posture as well.

While these hypotheses are compelling, they are largely based on single static or, at best, standing‐sitting radiographs. Even though the null hypothesis of this study is statistically robust, the absence of a correlation between reduced spinopelvic mobility and knee flexion deformity does not imply absence of their interaction in dynamic contexts such as gait. Ultimately, as with knee kinematics, such static assessments may not sufficiently explain the multifactorial, dynamic biomechanical interplay that is gait and posture [12].

An image‐based robotic platform was used to evaluate KFD due to its high accuracy and capability to measure dynamically in two scenarios: Once in the native state before removal of osteophytes and once after removal of osteophytes and with maximum manual corrective force. The accuracy of this image‐based robotic platform has been shown to be within 1.3 degrees of CT measurements for limb alignment; [30] this accuracy is considered superior to manual measurements with a goniometer or measurements made on smartphones [29]. Furthermore, robotic systems have demonstrated high repeatability and reproducibility in the assessment of soft‐tissue parameters [28]. Additionally, all measurements were performed under anaesthesia, thereby eliminating the confounding effects of pain on knee position.

The combination of robotic systems with modern alignment philosophies, such as functional alignment, has been described as a reliable approach for addressing knee flexion deformity in patients undergoing TKA [15, 22, 27]. Nevertheless, high inter‐individual variability in ligamentous laxity indicates that not only bony, but also soft‐tissue anatomy, plays a role in pre‐ and postoperative knee range of motion [7]. Ultimately, stratifying by both bony parameters and by soft‐tissue laxity, for example, using the robotic evaluation of articular laxity (REAL) classification, may allow further personalisation in TKA [2, 6, 9, 10, 13]. In the present study, a statistically significant difference in preoperative knee flexion deformity (KFDb) was observed among CPAK types, with patients classified as CPAK type III demonstrating significantly higher KFDb compared to those with CPAK type I. However, a plausible biomechanical explanation for this finding could not be identified based on the available data.

Due to the absence of patient‐reported outcome measures (PROMs) in our study, we were unable to assess the clinical significance of our findings regarding patient satisfaction but plan to report on these in a follow‐up study. Furthermore, in the absence of follow‐up data, it remains unclear whether intraoperative KFD has a lasting impact on postoperative KFD, potentially resulting in a recurrence of KFD in patients who exhibited increased intraoperative values. Finally, subgroup analyses did not achieve sufficient power to allow for conclusions regarding statistically significant differences in KFDb and KFDa between SM groups, CPAK types VII and IX and FKP‐types.

Several recent studies investigated the knee‐hip‐spine axis [5, 14, 19, 21, 32]. However, to the best of our knowledge, our study is the first to report on the correlation between spinopelvic mobility and intraoperative knee flexion deformity in patients undergoing TKA. To further elucidate the interplay between lower limb alignment and its changes following TKA, future studies should investigate gait and posture using dynamic modalities such as gait analysis. Pairing these findings with PROMs will be essential to fully understand how these biomechanical interactions affect functional outcomes and patient satisfaction.

## CONCLUSION

Reduced spinopelvic mobility is not associated with increased intraoperative knee flexion deformity in patients undergoing TKA, suggesting that the static increased knee flexion observed in these patients is likely a dynamic rather than a permanent compensatory mechanism. This suggests that routine preoperative assessment of spinopelvic mobility may not be necessary in the context of TKA, as intraoperative correction of knee flexion deformity appears feasible even in patients with reduced spinal mobility.

## AUTHOR CONTRIBUTIONS

Lorenz Pichler, Rauf Alizada, Lea MS Cordes, Kerem Basarir, Asim Kayaalp, and Reha Tandogan have substantially contributed to conducting the underlying research and drafting this manuscript.

## CONFLICT OF INTEREST STATEMENT

The authors declare no conflict of interest.

## ETHICS STATEMENT

The study protocol was approved by the local Ethics Committee for Clinical Studies (No: 2/2024, 11 November 2024), and the study was conducted in accordance with the Declaration of Helsinki. Written patient consent was retrieved from all patients included in the study.

## Supporting information

Supporting information.

## Data Availability

The data that support the findings of this study are available from the corresponding author upon reasonable request.
