# Supplementary material for: Reduced spinopelvic mobility does not correlate with knee flexion deformity in patients undergoing total knee arthroplasty
Source: Knee Surg Sports Traumatol Arthrosc. 2025 Sep 9;34(4):1346–53. doi: 10.1002/ksa.70047 (PMC13037340; doi:10.1002/ksa.70047)
Supplement: Supplementary file 1 — Supporting information. [file KSA-34-1346-s001.docx]

| **Supplement 1, Distribution of functional knee phenotypes** | | |
| --- | --- | --- |
| **Functional Knee Phenotype** | **n** | **%** |
| VAR_HKA_6° + VAL_FMA_3° + VAL_TMA_3° | 39 | 14.4 |
| VAR_HKA_6° + VAL_FMA_3° + NEU_TMA_0° | 36 | 13.3 |
| VAR_HKA_6° + NEU_FMA_0° + VAL_TMA_3° | 34 | 12.5 |
| VAR_HKA_6° + VAL_FMA_6° + VAL_TMA_3° | 18 | 6.6 |
| VAR_HKA_6° + VAL_FMA_3° + VAL_TMA_6° | 16 | 5.9 |
| VAR_HKA_6° + NEU_FMA_0° + NEU_TMA_0° | 15 | 5.5 |
| VAR_HKA_6° + VAL_FMA_6° + NEU_TMA_0° | 14 | 5.2 |
| VAR_HKA_6° + VAL_FMA_6° + VAL_TMA_6° | 12 | 4.4 |
| VAR_HKA_3° + VAL_FMA_3° + NEU_TMA_0° | 10 | 3.7 |
| VAR_HKA_6° + NEU_FMA_0° + VAL_TMA_6° | 9 | 3.3 |
| VAR_HKA_3° + NEU_FMA_0° + NEU_TMA_0° | 7 | 2.6 |
| VAR_HKA_3° + NEU_FMA_0° + VAL_TMA_3° | 7 | 2.6 |
| NEU_HKA_0° + VAR_FMA_3° + NEU_TMA_0° | 6 | 2.2 |
| NEU_HKA_0° + NEU_FMA_0° + NEU_TMA_0° | 5 | 1.8 |
| VAR_HKA_6° + VAL_FMA_3° + VAR_TMA_3° | 5 | 1.8 |
| VAR_HKA_6° + VAR_FMA_3° + VAL_TMA_3° | 5 | 1.8 |
| NEU_HKA_0° + NEU_FMA_0° + VAR_TMA_3° | 4 | 1.5 |
| VAR_HKA_3° + NEU_FMA_0° + VAR_TMA_3° | 4 | 1.5 |
| VAR_HKA_3° + VAR_FMA_3° + NEU_TMA_0° | 3 | 1.1 |
| VAR_HKA_6° + VAL_FMA_6° + VAR_TMA_3° | 3 | 1.1 |
| VAL_HKA_3° + NEU_FMA_0° + VAR_TMA_6° | 2 | 0.7 |
| VAL_HKA_6° + VAR_FMA_6° + VAR_TMA_3° | 2 | 0.7 |
| VAR_HKA_3° + VAL_FMA_3° + VAR_TMA_3° | 2 | 0.7 |
| NEU_HKA_0° + VAL_FMA_3° + VAL_TMA_3° | 1 | 0.4 |
| VAL_HKA_3° + NEU_FMA_0° + NEU_TMA_0° | 1 | 0.4 |
| VAL_HKA_3° + NEU_FMA_0° + VAR_TMA_3° | 1 | 0.4 |
| VAL_HKA_3° + VAR_FMA_3° + NEU_TMA_0° | 1 | 0.4 |
| VAL_HKA_3° + VAR_FMA_6° + VAL_TMA_3° | 1 | 0.4 |
| VAL_HKA_6° + VAL_FMA_3° + NEU_TMA_0° | 1 | 0.4 |
| VAL_HKA_6° + VAL_FMA_6° + VAR_TMA_6° | 1 | 0.4 |
| VAL_HKA_6° + VAR_FMA_3° + NEU_TMA_0° | 1 | 0.4 |
| VAR_HKA_3° + VAL_FMA_3° + VAL_TMA_3° | 1 | 0.4 |
| VAR_HKA_3° + VAL_FMA_6° + NEU_TMA_0° | 1 | 0.4 |
| VAR_HKA_3° + VAL_FMA_6° + VAL_TMA_3° | 1 | 0.4 |
| VAR_HKA_3° + VAR_FMA_3° + VAL_TMA_3° | 1 | 0.4 |
| VAR_HKA_6° + NEU_FMA_0° + VAR_TMA_3° | 1 | 0.4 |
